# Animal Grafting

**Published:** 1867-12

**Authors:** 


### ANIMAL GRAFTING.

Mr. Best, a French naturalist, cut off a rat's tail and joined it on to the freshly cut tail of another rat; the parts grew together and waggled like any other tail; in four months it was cut in two at the point of union, when it was found that all the proper vessels, veins, arteries, and nerves were supplied. Dr. Allen, of Bond street, narrates that a tooth freshly drawn from a negro boy was inserted into the socket from which his mistress had a "snag" taken, and it grew firm and did her good service for many years. It was considered at that time, that as the boy belonged to the mistress, so did his tooth and that hence the exchange was no robbery.

But the tables, later on, were turned against the white man. The owner of a plantation had a faithful and most truthful old negro, to whom he committed the fattening of a turkey for a christmas dinner; but on christmas morning the turkey was no where to be found; some of the other "hands," on being questioned, gave the information, that the faithful old servant had killed the turkey the night before and made a good supper of it himself. The master was surprised and greatly grieved; and summoning the old man to his presence, he acknowledged that he had killed and eaten the turkey. But, said the master, don't you think it wrong to steal from me that way, making use of my property? Why no, massa, that's no stealing. I'm your property and the turkey was your property; here I am, and the turkey is in me, and we bofe belong to you as before, only turkey has changed places, and instead of being in the coop, he is in a safer place, in my stomach, and we bofe are yours, turkey and negro too and you have as much as you had before, so I done no wrong." The logic puzzled the master, and he let Sambo off.

